# Borderline Personality Traits and Emotion Regulation Strategies in Adolescents: The Role of Implicit Theories

**DOI:** 10.1007/s10578-021-01169-8

**Published:** 2021-04-29

**Authors:** Jane McLachlan, Mani Mehdikhani, Beth Larham, Luna C. Muñoz Centifanti

**Affiliations:** 1 Department of Clinical Psychology, University of Liverpool, Whelan Building, Brownlow Hill, Liverpool, L69 3GB UK; 2 Liverpool Heart and Chest NHS Foundation Trust, Thomas Drive, Liverpool, L14 3PE UK; 3 Salford Primary Care Psychological Therapy Service, Greater Manchester Mental Health Trust, St James House, Pendleton Way, Salford, M6 5FW UK; 4 FRESH CAMHS (Liverpool), Alder Hey Children’s NHS Foundation Hospital, Catkin Building, Alder Road, Liverpool, L12 2AP UK; 5 Department of Primary Care and Mental Health, University of Liverpool, Whelan Building, Brownlow Hill, Liverpool, L69 3GB UK

**Keywords:** Borderline personality, Adolescents, Rumination, Cognitive reappraisal, Implicit theories

## Abstract

Borderline Personality Disorder (BPD) is associated with emotion dysregulation. The emotion regulation strategies people adopt may depend on one’s belief about emotion as either fixed or changeable (termed “implicit theory”). We test this experimentally by modifying beliefs about emotions using virtual reality (VR). A sample of 29 adolescents (ages 14–17 years) were recruited from two adolescent inpatient units for an uncontrolled pilot trial of a new brief intervention. We measured BPD traits, beliefs about emotion, treatment preference, cognitive reappraisal and rumination, before the VR game and 14–31 days later. Adolescents with higher levels of BPD traits endorsed fixed beliefs of emotion and reported higher levels of rumination and lower levels of cognitive reappraisal. After a one-time message delivered via VR, adolescents evidenced an increase in belief that their emotions were changeable. These findings suggest beliefs about emotions may have an important role in interventions for adolescents with BPD traits.

**Supplementary Information:**

The online version of this article (10.1007/s10578-021-01169-8) contains supplementary material, which is available to authorized users.

Borderline Personality Disorder (BPD) is characterised by difficult interpersonal relationships, emotional instability, and impulsivity, including non-suicidal self-injury (NSSI; 1–3]. Adolescence is often marked by one or more of these features, but for most people these traits do not cause significant levels of distress [4]. However, for some, these traits can be associated with a poor quality of life [5]. Many features of BPD manifest during adolescence or young adulthood [6]. The majority of young adults with mental health difficulties first received any mental health diagnosis in adolescence [7, 8], which suggests that recognising and understanding BPD traits in adolescence may inform the development of early interventions appropriate to this population.

People’s beliefs about emotions may be a factor that plays a role in how they respond to negative affect. Implicit theories are beliefs that a person has about the inherent malleability of certain domains, such as emotions [9]. Individuals fall along a continuum of how they view controllability of emotions [10]. At one end, a changeable belief about emotion views this as dynamic and controllable through effort. At the other extreme, a fixed belief views emotion as unchangeable and immutable once they occur. Whether people lean towards a fixed or changeable theory of their own emotion has significant implications for emotion regulation tendencies [11].

People who endorse a fixed belief may think that changes cannot be made through exerting additional effort and thus are more likely to use emotion regulation strategies that are deemed unhelpful (e.g. rumination; [12]). Conversely, those who view emotions as malleable tended to use strategies that are considered adaptive in many settings (e.g. cognitive reappraisal; 11).

To our knowledge, prior research is non-existent on implicit theories of emotion within an adolescent clinical population. This is surprising given that adolescence is a crucial stage of development for early intervention; it is an opportunity to reduce chronicity, improve outcomes and prevent stigmatising labels in later life [13, 14].

Rumination is one of the most widely researched cognitive emotion regulation strategies and has been linked to BPD across a range of studies [15–18]. Rumination has also been associated with many of the destructive behaviours in BPD, such as NSSI [19], suicide attempts [20] and substance misuse [21]. Such ‘dysregulated behaviours’ [20] are difficult to control and can lead to impairment in a person’s daily and interpersonal functioning.

People with a diagnosis of BPD have been found to engage in cognitive reappraisal less frequently, although this strategy may reduce their emotional lability [22, 23]. People who use cognitive reappraisal interpret events in ways that can reduce the negative impact of the emotional response [24, 25] and this has been shown to be a protective factor from psychopathology [26]. There is limited research on the longitudinal development of cognitive emotion regulation strategies, but initial findings indicate a strong linear increase in their use across the lifespan [27]. Adolescence therefore may be a crucial period for intervention to reduce use and impact of rumination and promote helpful strategies such as cognitive reappraisal that support positive wellbeing. What leads adolescents to show preference for one strategy over another remains unclear.

Finding ways to involve young people in treatment is key for early intervention. Historically, the retention of people with BPD in psychological treatments has been low, and the presence of BPD in clinical samples often predicts dropout [28]. Studies often do not report on how people engage with treatment. Results from treatment trials indicate that Dialectical Behaviour Therapy (DBT) and psychodynamic approaches are shown to be most effective for BPD, but effect sizes are low [29]. For people to initiate attempts to regulate their emotions, they must first believe that emotions can in principle be controlled and most importantly, that they can personally control them [30].

Virtual reality (VR) can be an innovative tool to enhance how we assess and treat mental health difficulties. VR involves the simulation of real-world experiences using computer graphics in which the user is immersed into and interacts with the virtual environment. It has been used in the treatment of specific phobias [31], social anxiety [32] and post-traumatic stress [33]. Emerging technologies such as VR and Augmented Reality (AR) have the potential to enhance how clinicians assess, diagnose and treat mental health conditions, including personality disorder. Potential benefits of VR (in research and in treatment) are ‘making the impossible, possible’ (to create environments which would be hard or impossible to recreate outside of the realm of imagination), ‘making the possible, safer’ (one can explore in complete safety dangerous or threat scenarios, for example in the context of exposure therapy) and by ‘making the possible safe, more practical and effective’ [34].

To our knowledge, no study to date has explored the use of VR for BPD within adolescent inpatient mental health services. Single-session interventions that promote a changeable belief of personality have shown to be effective in reducing risk factors for youth internalising disorders [35]. Thus, we used a single-session VR game as a platform for delivering a psychoeducational message on the changeability of emotions in inpatient settings.

In the present study, our aims were fourfold: [1] adolescents who endorsed more fixed beliefs of emotions would be more likely to report engaging in unhelpful cognitive emotion regulation strategies, such as rumination as compared to those who reported a more malleable belief of emotions. We also predicted that they would be less likely to report engaging in more helpful antecedent cognitive emotion regulation strategies, such as cognitive reappraisal. [2] We predicted that those with higher BPD traits would report more fixed beliefs than changeable beliefs, given the associated cognitive and behavioural difficulties that those adolescents experience. [3] Adolescents would be more likely to report feeling that their emotions were more malleable rather than fixed after a period of 2–4 weeks following game play in VR where they were told a story which asserted the changeability of emotion. [4] There is a possibility that adolescents who hold a more fixed belief of emotion would be less motivated to engage in psychological therapies, since they might not believe that changing emotions is within their control. This possibility was explored.

## Methods

### Participants and Procedure

Participants were invited to take part in an uncontrolled pilot trial of a new brief intervention. Participants were drawn from all admissions to two National Health Service (NHS) inpatient child and adolescent mental health units in the North West of England. Exclusion criteria were those patients with a diagnosis of a learning disability or a current episode of psychosis. Sample characteristics are presented in Table 1. All study procedures were approved by the University of Liverpool and the NHS. We obtained written informed consent/assent from all the participants. Consent from a parent or guardian was obtained for those under the age of 16 years.

**Table 1:** Sample characteristics

Variable	Time 1 *n* (%)	Time 2 *n* (%)
Participants	30	19
*Sex*		
Male, n	9	6
Female, n	20	12
Transgender male, n	1	1
Age, mean (SD)	15.9 (1.2)	15.7 (1.3)
*Ethnicity*		
White, n (%)	25 (83%)	15 (78%)
Mixed race, n (%)	2 (7%)	2 (11%)
Asian, n (%)	1 (3%)	0 (0%)
Undisclosed, n (%)	2 (7%)	2 (11%)
*Current treatment*		
Medication, n (%)	5 (13%)	3 (16%)
Psychological therapy, n (%)	6 (20%)	3 (16%)
Combination, n (%)	17 (57%)	10 (52%)
Monitoring only, n (%)	3 (10%)	3 (16%)
*Primary psychiatric diagnosis*		
Autism Spectrum Disorder (%)	4 (13%)	3 (16%)
Adjustment Disorder (%)	1 (3%)	0 (0%)
ADHD (%)	2 (7%)	1 (5%)
Depression (%)	2 (7%)	2 (11%)
Eating disorder (%)	1 (3%)	1 (5%)
Previous psychotic episode (%)	3 (10%)	2 (11%)
PTSD (%)	2 (7%)	2 (11%)
OCD (%)	1 (3%)	0 (0%)
*No psychiatric diagnosis* (%)	14 (47%)	8 (42%)

*SD* standard deviation

Participants completed four self-report measures and then completed the VR task. After a minimum of 2 weeks (M = 19.42 days; SD = 7.14; Range = 14 – 31 days), all participants were invited to repeat the set of self-report measures. An option was provided to repeat the VR game if they chose, but this was at the end of all procedures as an incentive to complete the follow-up assessment. All participants received a £3 voucher as gratitude for their time at both time points.

### Measures

#### VR task

The VR game ran on a Blade Pro-17.3″ (full HD) laptop, with Core i7-7700HQ Processor with Hyper-Threading 2.8 GHz, a graphic card NVIDIA GeForce GTX 1060 (6 GB GDDR5 VRAM) and Windows 10 Home operating system. The immersive virtual environment was displayed on an Oculus Rift VR HMD DK2 system. The Oculus head mounted display provides an immersive 3D virtual environment in a wide field of view. The InMind version 1 VR game was designed by Nival [36] and was played on Steam. Participants were verbally introduced to a character that struggled with intense emotions but had recently discovered research findings that supported the notion that emotions are changeable (Appendix S1). The concept of doing things differently to help reduce intensity of negative experiences was reinforced via the VR game where they actively participated and took charge of changing emotions within the brain. Participants were asked to focus on red neurons and were told to “fire” on them to transform them to green, which signalled a reduction in the intensity of emotion. This was synonymous with reducing the intensity of an emotional experience by doing something differently. Participants completed a self-report measure of flow following their gaming experience. The participants’ flow experiences were measured using the Flow Short Scale [37]. Participants rated 10-items on a Likert scale from 1 (not at all) to 7 (very much), which assessed fluency in action and being absorbed by action.

#### Borderline Personality Traits

Borderline personality traits were assessed using the Borderline Personality Features Scale for Children (BPFS-C; [38]). Participants rated 24-items using a Likert scale from 1 (not at all true) to 5 (always true). Items 1, 5, 23 and 24 were reverse scored.

#### Implicit Theories of Emotions

Beliefs about emotions were measured using the Implicit Theories of Emotion Scale [10, 11]. Participants rated the degree to which they agree or disagree to 8 statements about the changeability of their own emotions or the emotions of others on a 5-point Likert scale. Changeability belief items were reverse scored and the mean was obtained. Higher scores indicated a fixed belief and lower scores indicated a changeable belief of emotion.

#### Emotion Regulation Strategies

Cognitive emotion regulation strategies were measured using the Cognitive Emotion Regulation Questionnaire (CERQ; [39]). Participants rated 36-items on a Likert scale ranging from 1 (almost never) to 5 (almost always). The items consisted of 9 subscales including rumination and positive reappraisal.

#### Hypothetical Treatment Choice

Single item adapted from [40]: “If you struggle, or were to struggle with emotional difficulties (e.g. uncontrollable outbursts of anger, intense sadness) and had a choice between some form of psychological intervention, medication, a combination of medication and psychological intervention or not treatment other than standard monitoring to help you with these difficulties, which would you choose?” Further information was given about what each treatment option would entail, and opportunity was provided for participants to ask the researcher.

## Results

### Data Analyses

Results from the G*Power calculations [41] for paired sample t-tests, assuming a *p* value = 0.05, a large effect size of 0.5, with a statistical power of 0.8, recommend a sample size of 27. One participant was excluded from analysis because they did not complete the VR task. This reduced the sample size at Time 1 to 29, and to 18 at Time 2.

Descriptive statistics are shown in Table 2. The z-scores for skewness and kurtosis indicated that only positive reappraisal at Time 2 had significant skewness and kurtosis, based on a z-score of larger than 1.96 for a sample size of less than 50 [42]. One outlier identified was readjusted to one score above the next highest score. No other normality issues were identified. To test engagement in the VR task, we examined the average score of flow. The average score for total flow (M = 48.03, SD = 11.20, range 28–70) was consistent with previous studies using the measure in community samples who reported a mean of 48.88 (SD = 10.90; Bian et al., 2016).

**Table 2:** Descriptive statistics for primary study variables at Time 1 and Time 2

	Time 2 (n = 18)			
	Cronbach’s *α*	Mean (SD)	Cronbach’s *α*	Mean (SD)
Rumination	0.884	3.534 (0.293)	0.695	3.597 (0.083)
Cognitive reappraisal	0.808	2.103 (0.241)	0.864	2.292 (0.254)
BPFS	0.794	3.478 (0.403)	0.798	3.382 (0.409)
Implicit beliefs—self	0.875	3.560 (00.325)	0.821	3.431 (0.404)
Implicit beliefs—General	0.798	3.190 (0.215)	0.645	3.014 (0.233)
Flow—fluency	0.811	4.874 (0.566)	0.655	4.630 (0.639)
Flow—absorption	0.270	4.698 (0.174)	− 0.274	4.583 (0.962)
Flow- Total	0.777	4.803 (0.443)	0.706	4.611 (0.732)

We used Spearman correlations to examine associations among measures at Time 1 and Time 2. We subsequently performed paired-samples t-tests to examine change from Time 1 to Time 2 to test the effect of the VR task which aimed to change beliefs about emotions.

### Does Implicit Theory of Emotions Relate to Emotion Regulation Strategies and BPD?

To test if people who held a more fixed rather than changeable belief of emotion were more likely to use higher levels of rumination and less cognitive reappraisal, we conducted Spearman’s Rho correlations (Table 3). The association between general implicit beliefs of emotion and cognitive reappraisal or rumination were weak and failed to reach significance.

**Table 3:** Spearman correlations between variables of interest at Time 1 and Time 2

	1	2	3	4	5	6	7	8
1.BPD Traits	0.696*	0.164	0.580*	− 0.054	0.189	− 0.172	0.339	− 0.356
2.Implicit beliefs—General	0.233	0.621*	0.042	− 0.101	0.007	0.264	− 0.196	0.033
3.Implicit beliefs—self	0.435*	0.453*	0.837*	− 0.593*	0.364	− 0.177	0.156	− 0.338
4.Cognitive reappraisal	− 0.477*	− 0.262	− 0.724*	0.810*	− 0.596*	0.106	0.071	− 0.152
5.Rumination	0.354	− 0.029	0.381*	− 0.384*	0.715*	− 0.071	0.113	0.315
6.Age (Time 1)	0.098	0.214	− 0.221	0.084	− 0.175	–	–	–
7.Gender (Time 1)	0.235	− 0.149	0.011	− 0.184	0.066	–	–	–
8.Psychiatric diagnosis (Time 1)	− 0.172	− 0.375*	− 0.304	0.147	0.189	–	–	–

Correlation coefficients for Time 1 are below the diagonal and Time 2 are above the diagonal, with covariance measures reported on the diagonal and in bold. Age, gender, and psychiatric diagnosis were only measured at Time 1

*p < .05

Similarly, we tested if people who held a more fixed belief of their own emotion were more likely to use more rumination and less cognitive reappraisal. Consistent with predictions, a more fixed belief of one’s own emotions was associated with higher levels of rumination at Time 1 (*r*_*s*_ = 0.38, *p* = 0.042); however, this did not hold at Time 2 (*r*_*s*_ = 0.37, *p* = 0.133). Cognitive reappraisal showed a strong and negative association with implicit beliefs of one’s own emotions at Time 1 (*r*_*s*_ = -0.72, *p* = 0.01) and a moderate positive association at Time 2 (*r*_*s*_ = 0.59, *p* = 0.01). Therefore, if adolescents considered their own emotions to be more fixed than changeable at Time 2, they reported using cognitive reappraisal during a negative experience.

We next tested if people with higher BPD traits would have higher fixed beliefs of emotion. A more fixed belief of one’s own emotions was associated with higher BPD traits (Time 1: *r*_*s*_ = 0.44, *p* = 0.018; Time 2: *r*_*s*_ = 0.58, *p* = 0.012). This moderate association suggests that adolescents with higher BPD traits also have a more fixed belief about their own emotion but not emotions in general (see Table 3).

Beliefs about emotions were not significantly associated with gender, age or ethnicity. Therefore, these variables were not included in further analyses.

Psychiatric diagnosis (coded as present = 1, absent = 0) was significantly associated with belief about emotion in general at Time 1 (*r* = -0.375, *p* = 0.045), but not at Time 2 (*r* = 0.033, *p* = 0.898). However, we explored this further through further analyses. Partial correlations controlling for psychiatric diagnosis for all variables were computed at Time 1 and Time 2. The significant associations remained for almost all variables, except for beliefs about one’s own emotion and emotions in general at Time 1 (*r* = 0.341, *p* = 0.071), suggesting that psychiatric diagnosis was masking this covariance. After controlling for psychiatric diagnosis, rumination and beliefs about one’s own emotions now showed a significant positive correlation at Time 2 (*r* = 0.501, *p* = 0.034). Further, the other significant findings remained significant: BPD still showed significant associations with beliefs about emotions and rumination but now rumination showed a positive association with BPD traits at Time 1 and Time 2, although only Time 1 was significant (*r* = 0.382, *p* = 0.041). Rumination still remained significantly associated with cognitive strategies and beliefs, and cognitive strategies remained significantly associated with beliefs about emotions, as before. Therefore, beliefs about one’s own emotions was associated with cognitive regulation strategies, regardless of psychiatric diagnosis.

### Do Implicit Theories of Emotion Change After Engaging in a Short VR Task?

The VR game was explicitly designed to intervene in people’s beliefs about the malleability of emotions. Based on a paired samples t-test, there was a significant increase in beliefs about the changeability of one’s own emotions from Time 1 to Time 2 in response to the VR game (*t* (17)  = 3.31, *p* = 0.004, *d* = 0.78). For completeness, we tested if adolescents would be more likely to adopt healthy cognitive emotion regulation strategies (i.e. cognitive reappraisal) rather than a more unhelpful strategy such as rumination after the VR game. To test this, we compared scores on the key variables before and after the intervention using paired samples t-tests. Results are shown in Table 4. Effects were small and did not reach significance for changes between the two time points for implicit beliefs about emotions in general, BPD traits, rumination and cognitive reappraisal. Thus, only the target of intervention showed improvement.

**Table 4:** Spearman correlations between variables of interest at Time 1 and Time 2 controlling for psychiatric diagnosis

	1	2	3	4	5	6	7
1.BPD Traits	0.681*	0.201	0.503*	− 0.119	0.311	− 0.197	0.407
2.Implicit beliefs—General	0.136	0.581*	0.053	− 0.139	− 0.030	0.186	− 0.186
3.Implicit beliefs—self	0.403*	0.341	0.805*	− 0.678*	0.501*	− 0.182	0.192
4.Cognitive reappraisal	− 0.474*	− 0.167	− 0.723*	0.834*	− 0.538*	0.184	0.041
5.Rumination	0.382*	0.057	0.463*	− 0.413*	0.709*	− 0.054	0.073
6.Age (Time 1)	0.035	0.229	− 0.292	0.152	− 0.145	–	–
7.Gender (Time 1)	0.240	− 0.210	− 0.004	− 0.189	0.079	–	–

Correlation coefficients for Time 1 are below the diagonal and Time 2 are above the diagonal, with covariance measures reported on the diagonal and in bold. Age and gender were only measured at Time 1

*p < .05

**Table 5:** Descriptive statistics for key variables and analysis of change from Time 1 to Time 2

Variable	Mean (SD)		Paired sample t-test			
	Time 1	Time 2	t	df	p	d
Implicit Beliefs -self	3.560 (0.965)	3.431 (0.835)	3.305	17	0.004	0.779
Implicit Beliefs—General	3.190 (0.850)	3.014 (0.597)	1.578	17	0.133	0.372
BPD Traits	83.483 (11.627)	81.167 (11.873)	0.21	17	0.836	0.05
Rumination	14.138 (4.711)	14.389 (3.381)	0.357	17	0.726	0.084
Cognitive reappraisal	8.276 (2.987)	9.167 (4.033)	− 1.661	17	0.115	− 0.391

## Does Implicit Theory of Emotion Affect Choice of Treatment for Emotional Difficulties?

Overall, a total of 3 participants (10.3%) indicated that they would prefer psychological therapies as a treatment option, 5 participants (17.2%) selected the medication only option, 17 participants (58.6%) opted for a combination of psychological therapies and medication, whilst 4 participants (13.8%) selected the ‘No treatment’ option at Time 1. At Time 2, only 1 participant (6%) opted for psychological therapies as a treatment option, compared to 3 (17%) who opted for medication or 12 (67%) who opted for a combination of psychological therapies and medication. A total of 2 (11%) participants indicated a preference for no treatment at time 2. Given the small numbers within each category and little change over time, we did not conduct further statistical testing.

## Discussion

The present study showed the effectiveness of a short VR task which aimed to modify the beliefs about the changeability of emotions for inpatient adolescents with BPD traits. In the present study, we found BPD traits were significantly associated with implicit beliefs about one’s own emotions, such that they felt they were unable to take control of their own emotions. Also, adolescents with more fixed beliefs about own emotions reported more rumination and less cognitive reappraisal. Finally, these beliefs about one’s own emotions being changeable increased over time after a one-time message through an active VR experience during which adolescents took control of emotions. Although based on a small sample, these findings suggest that interventions based on modifying beliefs about the malleability of emotions could be beneficial when using a game in VR. Virtual reality may be an engaging tool for adolescents in inpatient mental health units, since the adolescents in the present study reported experiencing “flow”.

We found that BPD traits were associated with beliefs about own emotions but not emotions in general. Young people’s beliefs about their own emotions are more pertinent for BPD traits than beliefs about emotions in general. This is supported by previous research looking at wellbeing and psychological distress in a community sample [11]. Beliefs about the self in relation to others are parallel with research into self-stigma amongst women with BPD [43]. In this context, self-stigma refers to the notion that they alone are inadequate and perceived as such from others. Self-stigma is shown to be inversely related to self-efficacy, which determines the effort an individual will expend [43]. People with high self-efficacy in a certain domain display more effort and persistence [44], which may bear some overlap with the implicit beliefs literature. Future research could consider applying the implicit beliefs framework to self-stigma of mental health difficulties, particularly BPD (Table 5).

Consistent with predictions, the present study showed that adolescents with stronger beliefs in a fixed theory of emotions were more likely to ruminate and less likely to use cognitive reappraisal to regulate their emotions. These results support previous findings amongst community samples [11, 45]. This is akin to the learned helplessness theory [46], which describes the passive behaviour a person may engage in if enduring a repeatedly painful experience that they are unable to escape or avoid. These results support the idea that a person’s perceptions of possibility to change one’s own attributes may influence the person’s use of strategies to change. If a person believes that they have no control or ability to influence the intensity of emotions as they occur, they are more likely to passively dwell on their negative mood.

Our findings have implications for treatment. Focusing on emotion regulation strategies raises the distinct possibility that promoting a changeable mindset may lead to reductions in symptoms. The main purpose of this study was to test if a brief changeable mindset intervention, delivered through a VR platform, increased the belief in the malleability of emotions. We also explored if the intervention reduced the use of rumination and increased the use of cognitive reappraisal in adolescents in a mental health inpatient unit, given that implicit beliefs about emotions were related to emotion regulation strategies. The results revealed that there was an increase in adolescents endorsing a changeable mindset of emotions whilst there was no significant change in the use of cognitive emotion regulation strategies between the two points of testing. These results are consistent with recent research in which students increasingly favoured a growth mindset of emotion following two 45-min intervention sessions [47]. The current study expanded on these findings by producing a change using a very brief and innovative mode of intervention. This has practical clinical implications for the use of such a tool in a range of settings, such as schools or mental health services, to explicitly deliver this message efficiently.

To our knowledge, the current study was the first to explore the effects of an intervention for implicit beliefs of emotion with adolescents in a clinical population. The lack of significant change in cognitive emotion regulation strategies may be attributable to the severity of mental health difficulties and thus how embedded these strategies might be within this population. The intervention in the current study explicitly addressed the possibility of change in emotions, which impacted their beliefs in this domain. It did not however, directly challenge their beliefs about the changeability of thinking styles, such as rumination, which may have led to little change in this domain. Further research may hope to explore interventions addressing various domains and the differential impact on a range of outcomes. Future research may determine if repeated exposure to the brief intervention produces any changes in cognitive strategies, treatment preferences and motivation to engage in treatment for a clinical population. This could be explored over a longer time period. With the data we collected on treatment preferences, we were unable to explore effects over time.

The results from this study should be interpreted in light of the following limitations. Firstly, a control condition was not included in which participants either received a message promoting a fixed mindset of emotions or treatment as usual. Naturally, this impedes any conclusions about specific mechanisms by which the intervention exerted its effects, over and above the effect of time. Furthermore, the sample population was receiving some form of treatment as part of their inpatient stay, we do not know if the intervention worked or if people changed their implicit beliefs because of treatment in the facility. Research has indicated that both psychological and psychiatric interventions may drive changes at a behavioural, functional, and neurological level [48–50] for psychotic and non-psychotic disorders. Many of the participants were receiving psychological and psychiatric interventions during their stay on the units. These treatments could have contributed to the change in the young people’s beliefs about the changeability of emotions between the two timepoints. Thus, without a control group we are unable to determine whether the VR intervention was the only mechanism of effect.

Psychiatric diagnosis was controlled for in the initial analysis via partial correlations, but not for changes between timepoints due to insufficient power. Thus, we do not know if those with a current psychiatric diagnosis have changed less (or even more) over time than those without a diagnosis. We were also unable to see if different diagnoses might be more or less amenable to this brief intervention. We urge future research to examine this possibility.

Further, girls were overrepresented in the present study so our findings may be more representative of clinical samples of girls. Indeed, girls are more likely to have BPD traits [52]. Because the zero-order correlations involving gender were nonsignificant, gender was not controlled for in further analyses. However, we had a higher proportion of girls, which may have driven the associations that we found, so the associations between BPD traits with regulation strategies and beliefs about emotions might have been more strongly positive than if there were greater numbers of boys.

We did not measure educational attainment and intellectual ability in the present study. The presence of internalising or externalising problems, such as those tied to BPD, has been associated with educational attainment [51], so intellectual functioning could affect the ability to take on board ideas about the controllability of emotions. This would be especially important, since our task involved listening and processing a story about a person dealing with emotions. This is why we had excluded known patients with IQ less than 75. We also did this to ensure participants could complete the questionnaires.

Finally, the small sample size means that some of the statistical analysis may be underpowered. Nonetheless, current work provides a foundation for future investigations into implicit beliefs of emotions in mental health; specifically, beliefs about the changeability of one’s own emotions may play an important role in presentation and treatment of BPD difficulties. Future research ought to track the progress of participants longitudinally, to determine if the onset of symptoms precedes the implicit beliefs.

Awareness of these limitations should not detract from the importance of the practical implications of the study. Notably for this study, the initial associations observed between a fixed belief of emotions, BPD traits and cognitive emotion regulation strategies indicates that these beliefs may have an important role to play in the aetiology and subsequent treatment of these difficulties. This research has enabled a timely integration of phenomena from social and educational psychology in a clinical psychology setting.

The present study's findings support the appropriate use of VR with this population. Our findings underscore the potential benefits of brief positive psychoeducational messages using this technology. Furthermore, the intervention used in the current study could help with the acquisition of skills to manage emotions in a non-clinical population. Of practical use to conducting research, in our experience, employing the use of VR facilitated the recruitment of adolescents. Although motivation to participate was not a focus of the present study, our anecdotal data supports the possibility of using VR to engage hard-to-reach groups in psychoeducational interventions.

## Summary

People’s *beliefs* about emotion as either fixed or changeable have been found to be associated with anxiety, depression and adjustment to major life transitions. However, very little work has been undertaken to investigate the influence of a person’s belief about emotions on emotion regulation strategies in a clinical population. The present study provides novel insights into the changeability of one's beliefs about emotion, which informs the emotion regulation strategy adopted. These beliefs may be targeted with interventions delivered via VR.

The results of this study suggest that after a one-time message via VR promoting the changeability of emotions, adolescents evidenced an increase in the belief that emotions were indeed changeable. A fixed belief of one’s own emotions was associated with higher Borderline Personality traits, higher levels of rumination and lower levels of cognitive reappraisal. These findings not only have implications for the treatment and management of emotion regulation difficulties but demonstrate the utility of VR with this clinical population. Promoting a changeable mindset raises the distinct possibility of reduction in symptoms. The use of VR highlights the benefits of brief psychoeducational messages delivered using digital technology to young people.

## Supplementary Information

Below is the link to the Supplementary Information.

Supplementary Information 1 (DOCX 1731 kb)
