# Supplementary material for: Borderline Personality Traits and Emotion Regulation Strategies in Adolescents: The Role of Implicit Theories
Source: Child Psychiatry Hum Dev. 2021 Apr 29;53(5):899–907. doi: 10.1007/s10578-021-01169-8 (PMC9470600; doi:10.1007/s10578-021-01169-8)
Supplement: Supplementary file 1 — Supplementary Information 1 (DOCX 1731 kb) [file 10578_2021_1169_MOESM1_ESM.docx]

## **Appendix S1**

This is Joe. Joe has always had strong feelings. When he was happy, he was really happy – running around, laughing and joking with his friends. When he was mad with someone, he could burst out in anger very quickly. When he was sad, he found himself getting really low quickly, was unable to shake it off and sometimes cried uncontrollably.
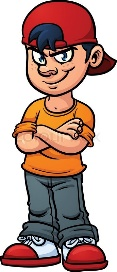


Joe wondered what was going on and whether he could control his feelings. He decided to find out.

It turns out that there had been recent research into feelings. Joe found that the research said feelings were dynamic and changeable. Researchers have looked at activity in the brain and the brain is changeable too. So, patterns of behaving translate into brain patterns but these patterns can be reversed through hard work. Researchers found that things, like what we do often, the activities we join in, the focus we have on managing our feelings and experiences we have over time, can affect how we feel about our feelings. This can then affect the way we experience feelings.

Joe thought about this research and what it meant to him.

Joe would like to take you on a journey through his brain to help him notice and manage these feelings. Help him to work hard to change those brain patterns so that his feelings can improve and feel more manageable. While you travel through, you will look for the parts of the brain (we call these neurons and they look like this (look at picture on left)) that are having strong feelings. You will know this because they will be glowing red. Joe would like you to help him do things differently and so not feel this emotion so strongly. You can help him by looking at the glowing neuron and firing at it. This is similar to a lot of psychological help that is out there. So, we know that talking therapies and medicines both can change the brain and the way it reacts to events. You have the power to change Joe’s brain to help him out.
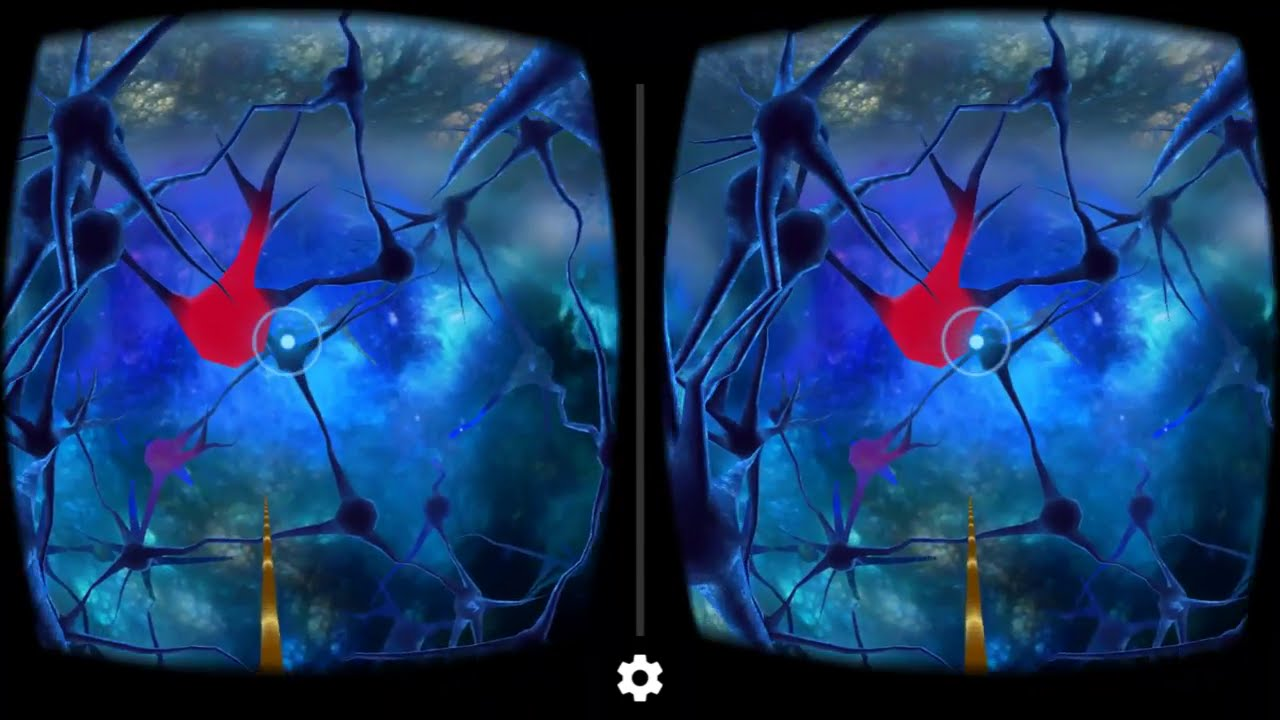


If you have any questions at any point or would like to stop. Please tell the researcher with you.
